# I don’t feel sick: Cognitive and affective processing of self-health associations using the Implicit Association Test

**DOI:** 10.1177/13591053241233509

**Published:** 2024-02-27

**Authors:** Eda Tipura, Isabele Jacot De Alcantara, Amélie Mantelli, Léa Duong Phan Thanh, Anna Fischer, Patrik Vuilleumier, Roberta Ronchi

**Affiliations:** 1 University of Geneva, Switzerland; 2 Georg August University of Göttingen, Germany; 3 Geneva University Hospitals, Switzerland

**Keywords:** EEG, implicit association test, implicit health, positivity bias, self-concept

## Abstract

Measuring implicit associations of self-concept with health or illness attributes may offer valuable insight into the mechanisms entailing the perception of one’s own health, as explicit measures of self-reported health are usually influenced by social desirability or response bias. In this study, healthy participants performed a modified version of the implicit association test (IAT) investigating implicit associations between the self and either health or illness related representations. Behaviorally, implicit associations dominated for self-health pairing, and their strength was inversely correlated with depressive traits. Neurally, concomitant EEG recording showed significant modulations of the P1, LPP, and N4 components evoked by such pairings, suggesting a facilitation of sensory responses to self-related stimuli and differential emotional processes engaged to integrate health versus illness information into self-related representations. These data offer new clues to better understand the cognitive and affective mechanisms underlying unrealistic optimism and pathological awareness of health conditions in various clinical populations.

## Introduction

Within the field of health psychology, clinicians, and researchers usually assess someone’s self-health perception, or self-reported health, with explicit questions (e.g. how is your health in general?) and qualitative response labels varying from excellent to poor (Jylhä, 2009). Although an explicit question provides valuable information on a patient’s current health and is a reliable indicator of their future well-being (DeSalvo et al., 2006), it is also limited as social desirability or response bias tend to influence the outcome being measured (Park et al., 2003; Werntz et al., 2017). It has therefore been suggested that implicit associations might be another interesting avenue to investigate how individuals perceive their own health in clinical or research settings (Werntz et al., 2017). One possible way to investigate these associations is to use the implicit association test (IAT), which was initially developed by Greenwald et al. (1998) and allows measuring the strength of automatic associations between concepts and attributes. For example, in a task where participants have to categorize words as either flowers or insects, as well as positive or negative affective attributes, the speed of categorization is consistently modulated by the strength of the association between the categories and attributes. When highly associated categories share the same response key (e.g. flowers and pleasant), participants tend to respond faster than when the categories have weaker associations (e.g. insects and pleasant). This task has been used to investigate implicit biases and the strength of associations in many areas of psychological research, including implicit associations of information related to the self (Greenwald et al., 2009), and the underlying neural mechanisms have been explored with brain imaging techniques such as electroencephalography. For example, using a personality IAT with self (vs non-self) stimuli, Fleischhauer et al. (2014) showed that the behavioral IAT effect was paralleled by an enhancement of the P1 and P3 components in concomitant EEG responses, which was interpreted as a facilitation of processing self-relevant stimuli. In their study, both early perceptual as well as later decision-related processes contributed to the IAT effect.

Noteworthy, social psychology studies also showed that individuals have a superior or enhanced memory for stimuli that relate to the self (Symons and Johnson, 1997). When participants make judgments about the self or others, self-referenced judgments are faster and more confident (Kuiper and Rogers, 1979). Self-processing also involves an attentional aspect, with self-related stimuli automatically capturing attention (Alexopoulos et al., 2012). In addition, it has often been reported that people exhibit a general tendency toward positively biased self-attributions (Mezulis et al., 2004). For example, using a priming paradigm, Chen et al. (2014) showed faster responses to self-related positive adjectives and other-related negative adjectives, relative to alternative pairings, indicating an implicit self-positivity bias. Accordingly, event-related potentials in EEG showed larger P300 amplitudes for words that disclose a self-positivity bias, suggesting that the pleasantness or agreeableness of self-related information receives more attentional resources. Furthermore, larger N400 amplitudes elicited by words that are inconsistent with the self-positivity bias suggest that accessing non-self-relevant information is more difficult than self-relevant information.

The P3 and N4 amplitudes measured by EEG could therefore be used as valuable neuro-indices of the implicit self-positivity bias. Using a self-esteem IAT to measure implicitly valenced self-processing, Grundy et al. (2015) reported that the IAT effect was associated with significant modulations of the N1, P2, P3, and LPP components. The self-negative condition generally displayed larger event-related potential amplitudes than the self-positive condition in both high and low self-esteem groups. These effects appeared earlier, were larger, and expressed more asymmetrically over the left hemisphere for the high self-esteem group, possibly reflecting differences in attentional resources devoted to teasing apart the two oppositely valenced associations of these words. Additionally, Wu et al. (2016) showed that the IAT effect modulated the N2 and P3 components: participants responded faster in a congruent condition (compared to an incongruent condition) in which “self” was paired with “positive” traits and “others” was paired with “negative” traits. The incongruent condition elicited a larger N2 and a smaller P3 compared to the congruent condition, which was taken to suggest that both executive control and stimulus categorization processes were engaged by implicit self-evaluation. Altogether, these findings show that multiple but heterogeneous processes are presumably involved in implicit self-perception, with some variability due to the tasks used and the nature of associations tested, and a role for both automatic and more controlled attentional processes that may differentially contribute to the type and magnitude of behavioral and/or neural effects.

A few recent studies sought to link the mechanisms of self-biases and self-processing to the concept of health. A study conducted by Werntz et al. (2017) extended the use of the IAT to assess implicit associations between categories of health- versus illness-related words and self versus other concepts across the lifespan. They found that their health-IAT provided a valid and reliable measure for assessing implicit self-concept linked to physical health, that is, participants responded quicker for congruent (me-health and other-sick) than incongruent (me-sick and other-health) trials. Importantly, this effect covaried with the age of participants, such that older participants formed stronger associations between self and health than younger participants. The researchers interpreted this age-dependent positive self-health relationship as possibly arising from older individuals comparing themselves to the stereotype of frail elderly individuals when rating their health. These data further highlight the need to better understand the cognitive and affective mechanisms underlying self-health representations, as they may influence health-related decision-making and behaviors and constitute an important factor to consider when evaluating patients’ perception of their disease in clinical or research settings.

The aim of the present study is to confirm the self-serving attributional bias linked to health (vs illness) related categories, and explore the brain mechanisms involved in this process by measuring EEG while participants perform a health-IAT. We hypothesize that healthy participants will show the expected congruent association between self and health, and that this will result in a modulation of specific brain potentials linked to self and emotion processing, such as the N2, P3, and LPP components. We will also examine potential associations between the self-health bias and individual personality and emotional characteristics.

## Materials and methods

### Participants

An a priori power analysis was performed using G*Power 3.1.9.4 (Faul et al., 2007) for a medium effect size of 0.25, using “F-tests,” “ANOVA: Repeated measures, within factors” and an alpha of 0.05. A total sample of 20 participants was required to achieve a power of 0.90.

We recruited 27 healthy adult participants using posters placed at the University of Geneva. Prior to the inclusion in the protocol, a customized health questionnaire was administered to each volunteer, to exclude participants with major and/or chronic illness that could affect their self-health perception. After inclusion, six participants were excluded because of high scores of anxiety/depression (⩾11) in questionnaires or discovery of an important health issue that was not declared during the pre-selection. Our final sample consisted of 21 participants, 14 males and 7 females with a mean age of 62.31 years (SD = 10.87, range 36–76). Participants were all right-handed (mean laterality coefficient = 93.42, SD = 10.77 (Oldfield, 1971)) and had a normal or a corrected-to-normal vision. In addition to the experimental procedure (see below), participants filled in the Hospital Anxiety and Depression Rating Scale questionnaire (HAD; Snaith and Zigmond, 1986), which is a self-reported scale consisting of 14 items exploring anxiety and depression symptoms during the past week. The highest score on each domain (depression, anxiety) is 21, and scores equal or above 11 are considered to reflect moderate levels of depression and anxiety. Our participants reported a mean of 2.81 (SD = 2.37; range: 0–9) on the depression scale and a mean of 4.9 (SD = 2.62; range: 1–10) on the anxiety scale. They also filled in the Life Orientation Test-Revised (LOT-R; Scheier and Carver, 1985; French version: Trottier et al., 2008) to explore dispositional optimism. The mean LOT-R score in our sample was 17.14 (SD = 4.16; range: 8–24). Finally, participants were also given a quality of life questionnaire (World Health Organization Quality of Life, WHOQOL-BREF; von Steinbüchel et al., 2006) assessing life quality in four domains: physical, psychological, social, environmental. For each sub-section, scores were transformed into a normalized range of 0–100, with a higher score representing a higher perceived quality of life. Our participants reported a mean of 82.57 (SD = 11.45; range = 56–100) on the physical domain, 76.19 (SD = 12.79; range = 38–94) on the psychological domain, 69.71 (SD = 12.57; range = 44–94) on the social domain, and 80.19 (SD = 14.75; range = 44–100) on the environmental domain.

Participants were paid 20 CHF per hour for their participation. The study was approved by the local ethics committee (University of Geneva) and was performed in agreement with the Declaration of Helsinki. All participants signed an informed consent form before participating.

### Stimuli and experimental procedure

All participants performed a health-IAT and a control-IAT. The tasks were programed and performed using E-Prime 2 software. In order to assess implicit associations between health/illness and self/other concepts, we created a 2 × 2 IAT with the concepts of illness versus health and self versus others. In this task, participants were placed in front of a computer screen and performed seven blocks of the task with a total of 180 trials in each task (see Supplemental Appendix B). Ten French words referred to the concepts of “Self” or “Others,” and 40 words referred to the “Health-Illness” categories (see Supplemental Appendix C). Specifically, 20 words strongly associated to health and 20 associated to illness were selected from a database of 80 words, matched for length and frequency, and validated by prior pilot test (in a different group, *n* = 33) showing higher ratings for their association to a specific concept and a significant difference (*p* < 0.01) for their association to the opposite concept.

We followed a standard IAT procedure with seven successive task blocks (Greenwald et al., 2003) (see Supplemental Appendices A and B). The first two blocks were training blocks. In the first block, participants had to classify a word (e.g. “me”; “their”) displayed in the center of the screen, according to its relation to the concept of either self or others (as indicated by labels at the top and bottom of the screen) using the computer keyboard (by pressing either the up- or down-arrow keys). In the second block, they now had to classify a word (e.g. “vitality”; “weak”) according to the concept of either health or illness. The third block represented a final training step where the concepts were combined. The concepts of self or others were respectively paired with either the concept of health or the concept of illness, by placing the two corresponding labels together at the top or bottom of the screen (same position as preceding blocks), while the central word was taken from one of these two categories. The same computer key (up or down) was now used for the paired concepts (at the top or bottom of the screen). This was followed by a repetition of the same task (fourth block) that served the proper experimental test for CONGRUENT associations (health-self vs illness-other).

The fifth block was a new training block with a reverse configuration using only the concepts of health and illness, but now presented with a different position on the screen (top or bottom) relative to the first two blocks. The sixth block trained this new configuration combined with concepts of self and others (shown at a similar position as in prior blocks). Lastly, the seventh block repeated the same conditions as the sixth and constituted the final experimental test for INCONGRUENT associations (illness-self vs health-others). The position (top/bottom) of the health/illness or self/other categories in the first and second blocks was counterbalanced across participants.

All participants also underwent a control IAT, unrelated to self or health concepts. This task followed the same block structure as the experimental IAT (Supplemental Appendix B), but with different semantic categories: health/illness terms were replaced by the concepts of positive/negative valence, and self/other terms by flower/insect words.

Both the experimental and the control IAT were counter-balanced in terms of task and test order (i.e. incongruent or congruent block first). We also balanced the association between the up/down buttons and categories.

### EEG acquisition

EEG was recorded using a 64-channel Biosemi Active-Two system (Amsterdam, Netherlands) with AG/AgCl electrodes positioned according to the extended 10–20 system. Four additional flat electrodes were placed on the outer canthi of the eyes and above and under the right eye, in order to capture eye movements and blinks. Each active electrode is represented with an impedance value, which was kept below 20 kΩ for each participant. The EEG was continuously recorded with a sampling rate of 1024 Hz. Data was re-referenced off-line against the average reference.

### EEG processing

Standard processing of EEG data was done offline using the software Brain Vision Analyzer V.2 (Brain Products, Gilching, Germany). The data were downsampled to 512 Hz and filtered between 0.1 and 30 Hz (order: 2). Bad electrodes were interpolated using a spherical spline (1% of the electrodes were interpolated). Eye movements and blinks were corrected (Gratton et al., 1983) and trials containing artifacts (automatic inspection; minimal allowed amplitude: -100 µV; maximal allowed amplitude: 100 µV) were removed (8%).

### Behavioral analyses

IAT data were analyzed using the scoring procedure developed by Greenwald et al. (2003). Reaction times (RT) were recorded from each trial and the algorithm calculated the IAT effect size (d’), reflecting the RT difference between the congruent and incongruent blocks (training and tests blocks). All RT above 10 seconds were excluded from the analysis. For errors (wrong word-category association), a penalty was applied where RT was replaced by the block mean RT of correct trials plus 600 ms. Only the third, fourth, sixth, and seventh blocks were considered for these analyses. After averaging the RT per block, two subtractions were made (incongruent – congruent RTs) for practice blocks and for test blocks separately. These two differences were divided by the pooled standard deviation of B3-B6 and B4-B7. We then averaged the two quotients to obtain the IAT effect (d’). A positive/high IAT effect (above 0.2) illustrates faster responses to the congruent associations and a negative/low IAT effect (below −0.2) illustrates faster responses to the incongruent associations. An IAT effect around 0 is considered as a “non-effect” (i.e. no notable difference in the subject’s latencies for congruent or incongruent associations). A perusal of the individual performance was done, to verify if each subject had a positive or a negative d’ effect. A single sample t-test was performed to test the group effect size of the d’ *(t*-test against 0).

Finally, Pearson correlations were performed between the d’, the LOT-R, the HAD-D HAD-A and the WHOQOL-BREF to test whether the IAT effect correlated with anxiety and depression levels, dispositional optimism, and the four domains of the quality of life questionnaire. We also tested whether the health-IAT effect correlated with age using a Pearson correlation.

### Electrophysiological analyses

#### Self and other related words

We performed stimulus and response locked ERPs. ERPs primarily focused on self and other related words only. Following visual inspection of grand averages, we assessed differences between congruent (self-health and other-illness) and incongruent (self-illness and other-health) conditions on the P1, N1, and LPP components time-locked to the stimulus, and the N4 component time-locked to the response. A collapsed localizer was used to determine the time windows for statistical analysis, based on the peaks and means observed in the grand averages across all conditions. Mean amplitudes were computed for each component. Following stimulus appearance and response, ERPs were computed from −200 to 800 ms using the 200 ms pre-stimulus period for baseline correction and the 200 ms pre-response period for response-locked ERPs.

The P1 mean amplitudes (110–170 ms) and the N1 mean amplitudes (160–240 ms) were extracted over electrodes on the left (P7, P9, PO7) and right (P8, P10, PO8) hemispheres. Repeated measures ANOVA were carried out for the P1 and N1 amplitudes separately with factors laterality (left/right), electrode (P7/P8, P9/P10, PO7/PO8), who (self/other), and pairing condition (congruent/incongruent) as within-subject factors. The LPP mean amplitudes (500–700 ms) were extracted over electrodes on the left (TP7, P7, P9), midline (CPz, Pz, POz), and right (TP8, P8, P10) hemispheres. A repeated-measure ANOVA was carried out for the LPP amplitudes with factors laterality (left/midline/right), electrode (TP7/CPz/TP8, P7,Pz,P8, P9/POz/P10), who (self/other), and pairing condition (congruent/incongruent) as within-subject factors.

Following response, ERPs were computed from −200 to 800 ms using the 200 ms pre-response period for baseline correction. The N4 mean amplitudes (250–400 ms) were extracted over electrodes on the left (P7, P9) and right (P8, P10) hemispheres. A repeated-measures ANOVA was carried out for the N4 amplitudes with factors laterality (left/right), electrode (P7/P8, P9/P10), who (self/other), and pairing condition (congruent/incongruent) as within-subject factors.

When necessary, adjusted p-values and degrees of freedom were used to control for sphericity (Greenhouse-Geisser correction).

## Results

### Behavioral results

#### Health-IAT

**Figure 1.**
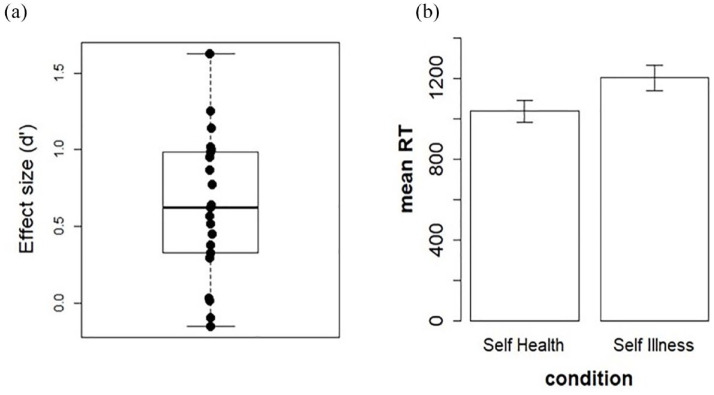
(a) Distribution of the effect size (d’) for the health-IAT. (b) Mean reaction times in the self-health condition (congruent) and self-illness condition (incongruent).

The average d’ for the health-IAT effect was of 0.62 (±0.47) (Figure 1(a)). The *t*-test against 0 on the d’ was significant: *t*(20) = 6.128, *p* < 0.05, showing that overall participants were highly susceptible to the IAT effect. This was confirmed by visual inspection of individual performances. In order to check for a specific self-effect, without a possible confounding effect due to the “others” attribution of health/ill categories, we extracted mean values of each participant for the two conditions including only the self (self/health and self/illness) and computed a paired t-test between these conditions. The t-test was again significant: *t*(20) = 4.33, *p* < 0.05, with shorter reaction times for the self-health than the self-illness condition (see Figure 1(b)).

The correlations between the health-IAT d’ and measures of optimism (*r* = 0.04), anxiety (*r* = −0.27), as well as the four domains of the quality of life (physical: *r* = 0.17; psychological: *r* = 0.29; social: *r* = 0.27; environmental: *r* = 0.23) were not significant (all *p*s > 0.05). However, the correlation between d’ and HAD-D was significant (*r* = −0.50, *p* < 0.05), with higher scores on this questionnaire (higher depressive traits) observed in individuals with a smaller health-IAT effect (d’). The correlation between the health-IAT d’ and age was not significant (*r* = 0.26, *p* > 0.05).

#### Control-IAT

The average d’ for the control-IAT effect was of 0.86 (±0.43). The t-test against 0 on the d’ was significant: *t*(20) = 8.99, *p* < 0.05, showing that on average participants were also highly susceptible to the IAT effect. Overall, these results show that both IAT tasks successfully probed implicit association biases in our participants for both health and non-health domains.

### EEG results

As mentioned in the method, our analysis focused on the P1, N1, and LPP components time-locked to the stimulus appearance, and the N4 component time-locked to the response.

#### P1 mean amplitude

**Figure 2.**
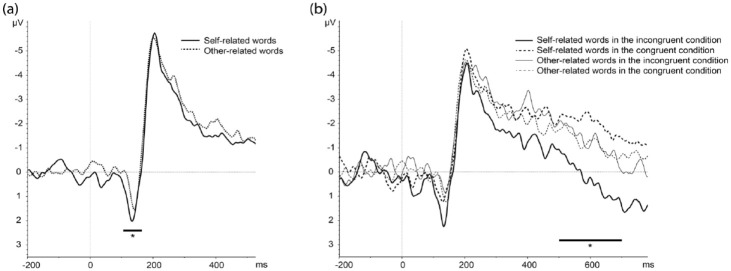
(a) Stimulus locked ERPs following self and other related words, showing a significant difference on the P1 component over the left hemisphere (P7, P9, PO7 pooled). (b) Stimulus-locked ERPs following self and other related words in the congruent (health-self and illness-other) condition and self and other related words in the incongruent (illness-self and health-other) condition, showing a significant difference in the LPP component over the left hemisphere in the latter case only (TP7, P7, P9 pooled).

The repeated measure ANOVA showed a significant interaction between laterality and the factor who (*F*(1, 20) = 9.16, *p* = 0.006, *η*^2^_*p*_ = 0.314). Post-hoc HSD Tukey tests indicated that self-related words yielded a higher amplitude than other-related words over the left hemisphere (*p* = 0.026) (see Figure 2(a)) but no difference over the right hemisphere.

#### N1 mean amplitude

No effects reached significance for the N1 mean amplitude (all *p*s > 0.05).

#### LPP mean amplitude

The repeated measure ANOVA showed a significant main effect of pairing condition (*F*(1, 20) = 6.99, *p* = 0.015, *η*^2^_*p*_ = 0.258), with incongruent trials leading to more positive amplitudes than congruent trials. Moreover, there was a significant interaction between laterality, pairing condition, and the factor who (F(1.58, 31.64) =4.77, *p* = 0.021, *η*^2^_*p*_ = 0.192). To better understand this three-way interaction, we performed distinct repeated measure ANOVAs for each laterality level (left, right, midline) using the pairing condition and who as within-subjects factors, which indicated that the who*pairing condition interaction was significant only for the left hemisphere (*F*(1,20) = 8.13, *p* = 0.009, *η*^2^_*p*_ = 0.289). Post-doc HSD Tukey tests then showed that incongruent trials produced higher mean LPP amplitudes than congruent trials for self-related words (*p* = 0.004), not for other related words (*p* > 0.05) (see Figure 2(b)).

#### N4 mean amplitude

The repeated measure ANOVA showed a significant main effect of pairing condition (*F*(1,20) = 9.92, *p* = 0.005, *η*^2^_*p*_ = 0.331), with responses to incongruent trials leading to more negative amplitudes than congruent trials. Moreover, there was a two-way interaction between pairing condition and electrode (*F*(1, 20) = 5.67, *p* = 0.027, *η*^2^_*p*_ = 0.221) as well as a triple interaction between the factor who, laterality, and electrode (*F*(1,20) = 5.11, *p* = 0.035, *η*^2^_*p*_ = 0.203).

## Discussion

The aim of the present study was to investigate the neural signature of a self-positivity bias associating the self with health representations, using a health-IAT paradigm probing automatic associations between health and illness related terms and the self-concept in a healthy adult population. First, at the behavioral level, we confirmed the expected presence of implicit associations between the self and health in participants with no major illness, as reflected by a large positive effect size measure (d’) in the IAT. This finding supports the results obtained by Werntz et al. (2017), albeit we found no modulation of this effect by the age of participants in our sample. Interestingly, we found a consistent result (i.e. strong association between self and health) when extracting RTs only from the self-related conditions, confirming that the self-health bias is present independently from any other effect modulating responses to the “other” category.

Interestingly, the magnitude of the IAT d’ correlated with subjective reports of depressive mood: the more participants disclosed strong associations between the self and health, the lower the presence of depressive-like symptoms. In contrast, there was no correlation with more general traits of optimism. In line with our findings, previous research has discovered that an implicit self-related bias concerning calmness versus anxiety was predictive of anxiety symptoms, whereas an implicit self-related bias concerning happiness versus sadness was predictive of depression symptoms (Werntz et al., 2016). Our study thus adds to the existing literature by suggesting a more general link between increasing levels of emotional distress and the implicit self-concept for health versus illness information, thereby emphasizing the role of affective processing on the integration of health representations with “self” features (or vice versa). Interestingly, depression symptoms have been associated with a differential perception of illness in various clinical contexts. For example, higher levels of depression correlate with the appraisal of the (negative) consequences of their physical illness in patients with rheumatoid arthritis (Murphy et al., 1999) or with the perception of illness status during the COVID-19 outbreak (Aqeel et al., 2022). Psychological distress has also been associated with illness perception in heart failure patients (Morgan et al., 2014). Overall, our results are in line with these findings by confirming that emotional distress may be closely interconnected, at an implicit level, with a feeling of being less healthy in more explicit, declarative, self-reports, even in a non-pathological adult population. This is consistent with some cognitive theories of depression (Beck, 1976) and the mood-state dependent hypothesis (Miranda and Persons, 1988), postulating that negative emotional states may “prime” negative self-representation in vulnerable people predisposed to emotional pathology. This suggests that dysfunctional beliefs and interpretations are associated with individuals’ emotional experiences. More broadly, these results accord with the notion that health and illness experiences and beliefs might be intimately grounded in cognitive and affective mechanisms responsible for constructing “models of the self” that may eventually determine both mental and physical well-being (Koban et al., 2021).

The IAT measures the strength of associations between opposite concepts and attributes. This might reflect a form of unrealistic comparative optimism (Shepperd et al., 2013), where individuals evaluate their own risks or likelihood of developing a disease as being lower than their peers, as the associations related to the self and the other concepts are inextricably linked and contrasted in the effect size measure. For example, people tend to underestimate their own risk of having fatal heart attack (Radcliffe and Klein, 2002), encountering alcohol problems (Dillard et al., 2009), or having breast cancer in the future (Gail et al., 1989), relative to the true average population risk. Three main causes of unrealistic comparative optimism have been proposed (Shepperd et al., 2015). First, people are motivated to maintain the belief that they are unlikely to encounter unfavorable outcomes. Second, people generally possess more information about the self than about others. Finally, this effect could be the consequence of the manner in which individuals process information. It has been shown that unrealistic optimism is associated with health consequences. For example, in one study individuals who exhibited unrealistic comparative optimism regarding the avoidance of the H1N1 virus had lower intentions to use hand sanitizers and wash their hands (Kim and Niederdeppe, 2013). Another study showed that middle-aged adults who were unrealistically optimistic had also limited knowledge about heart attacks and reduced recall of an essay they previously read on the risk factors associated with heart attacks (Radcliffe and Klein, 2002). This may delay seeking appropriate health care and reduce benefits from prescribed treatments. However, the lack of correlation between the health-IAT d’ and our measure of dispositional optimism (LOT-R questionnaire) suggests that this general measure of optimism is distinct from unrealistic comparative optimism associated with health representations. Optimism can be defined as “*a set of beliefs that leads people to approach the world in an active manner*” (Burke et al., 2000: 129). It therefore encompasses broader aspects than those implicated in the appraisal of health or illness, and may not be an optimal measure to capture self-related associations with categories used in the health-IAT task. Alternatively, the lack of correlation between dispositional optimism and the d’ could reflect the discrepancy between implicit and explicit measures of optimism, as proposed by Greenwald and Banaji (1995). As such, self-reported questionnaires reflect an explicit mode susceptible to social desirability while implicit measures activate more automatic responses, therefore relying on distinct processes. The LOT-R questionnaire used in this study is an explicit measure of optimism, which does not allow the investigation of automatic processes (Schnabel et al., 2006), and might therefore not correlate with reaction times measuring implicit associations.

At the neural level, we found that the self-health effect was underpinned by differential brain responses at several successive processing stages. ERP amplitudes revealed a differentiation between self- versus other-referential stimuli already at a very early perceptual stage, with enhanced P1 over the left hemisphere for self-related words. This effect might reflect sensory facilitation for self-relevant information. In the literature, the P1 component has been linked to the rapid and automatic perceptual analysis of visual stimuli. It is highly sensitive to attentional factors and provides a sensitive marker for an initial neural amplification of visual inputs by goal-directed attention, operating at an early stage of visual processing in the extrastriate visual cortex (Hopf et al., 2002; Luck et al., 2000). Previous work also found both early and late modulations of ERPs related to self versus other processing (Pinheiro et al., 2016), affecting N1, P2 and LPP components. In our study, we extend this self-processing advantage to the visual P1. This early functional impact of implicit associations in a health-IAT therefore points to more efficient categorization of words from the self and the other category. Information associated with the self-concept might be prioritized rapidly and independently of the semantic category (health or illness) and thus induce greater attentional capture and greater perceptual facilitation as compared to information associated with others. The left lateralization of this effect might reflect the verbal nature of the task, as already suggested by O’Toole and Barnes-Holmes (2009).

The integration of self-concept with health perception also affected later stages of stimulus processing. The LPP showed an interaction between the pairing condition (congruent vs incongruent) and the factor who (self vs other), with self-related words leading to more positive amplitudes when paired with illness than health terms. The absence of a similar (congruency) effect for associations with others suggests that this differential response (health vs illness) selectively modulated word stimuli when they were self-referential. This integration of health-illness terms with self concepts might involve an affective component, as the LPP has often been associated with emotional processing, with negative material typically leading to enhanced positivity of this component compared to neutral or positive material (Hajcak et al., 2009). These emotional differences were restricted to the left hemisphere in a previous study using words (Zhang et al., 2014), which could reflect heightened activity in the left fusiform cortex - an area showing selective responses to visually presented words (McCandliss et al., 2003). A larger LPP component was also observed by Zhang et al. (2020) when participant’s names were paired with negative words, as compared to when they were paired with neutral words. These findings accord with our own, showing a preferential interaction of self-relevance with health-related terms over the left hemisphere. Moreover, another IAT study using positive-valenced stimuli (baby and romance) and negative-valenced stimuli (snake and spider) also showed that incongruent trials lead to a more positive amplitude of the LPP than congruent trials at around 400–600 ms post-stimulus (O’Toole and Barnes-Holmes, 2009). In light of this literature, our results provide novel evidence that self-processing linked to health-related issues might have an affective, and not only cognitive, component, with stronger and/or negative valence associated with illness when related to the self. More generally, our results show that the enhancement of LPP occurred on incongruent trials, relative to congruent trials, that is when comparing self-illness with self-health trials. In this context, self-referential processing of illness terms might be accompanied by higher emotional arousal and activate threat/fear responsive mechanisms, triggering a more positive LPP. Future research should address whether this differs or not from mechanisms of unrealistic comparative optimism, which might instead reflect a protective mechanism to thwart the implicit threat evoked by the association between “me” and “illness,” and thus resolve a potential response conflict or dissonance at the cognitive level. In our study, no relationship was found between the IAT effect size and optimism scores on the LOT-R questionnaire, which, as noted above, may not reflect comparative optimism.

Finally, the main effect of congruency on the LPP extended to the response-locked N4 component. To our knowledge, response-locked ERPs have not been investigated in the context of an IAT. In other experimental contexts, stimulus-locked ERPs tend to show a larger N4 for incongruent than congruent trials (Williams and Themanson, 2011), suggesting that assessing self-incongruent information is more difficult than accessing self-congruent information as it requires more attentional resources.

### Limitations

The present study presents with some limitations, to be addressed by future research. At first, the lack of correlation between the d’ and the age of participants, or our measure of optimism, could be due to the sample size; this study would therefore benefit from replication in a larger sample. Moreover, as a methodological limitation it should be noted that the role of the “other” category in an IAT has been questioned. In a study using a self-esteem IAT (Karpinski, 2004), the valence of an unspecified other has been defined as negative. On the other hand, Pinter and Greenwald (2005) showed that in the IAT the valence of the other is near neutral in their studies. Therefore, while the self can be easily associated with positive attributes, the status of an unspecified other is less clear, and it should not automatically be considered as negatively valenced. Further investigations should consider different types of “others” to clarify the conditions in which an implicit bias appears.

## Conclusions and future directions

In summary, our study demonstrates that the processing of health related information discloses robust and implicit association biases preferentially linking the self with health versus illness concepts, and that this is underpinned by specific neural markers. This construction of self-features around the concept of health may involve both attentional and emotional components, and correlates with more general mood experience, such that the self-health bias is weaker in individuals with the tendency to have increased mood depression scores. At the electrophysiological level, we found distinctive brain responses discriminating the self- from the other-concepts at early perceptual stages (P1), while the interaction between health meaning and self-identity arose at later stages (LPP), possibly associated with emotional processing, suggesting that integrating the self-concept with (incongruent) illness cues may trigger negative affective appraisals and subjective threat.

Our findings may have important clinical implications. At first, it would be interesting to assess how true physical illness can affect and modify this self-implicit processing in the case of acute or chronic disease. Noteworthy, the emotional and cognitive factors of illness perception have recently been integrated in a model as a mediator between patient’s satisfaction and medication adherence in patients with rheumatoid arthritis and ankylosing spondylitis (Temeloglu Sen et al., 2023). It would be interesting to test whether implicit associations also play a role in this model. Moreover, we know that patients with neurological damage can present with a compromised awareness of having a disease, a condition called anosognosia (Babinski, 1914). As these patients may explicitly state that they are not sick or do not have specific symptoms, despite major neurological symptoms (e.g. hemiplegia), this task might allow probing if some implicit level of processing is still preserved, at the behavioral and/or neural level. In addition, the affective dimension of the health IAT effect indirectly inferred in our study may offer an interesting avenue for future research seeking to better characterize the dysfunctional or preserved mechanisms involved in pathological awareness of illness in various clinical populations. These new findings can be used to help patients to modulate maladaptive mechanisms facing the illness: brain stimulation techniques or neuro-feedback approaches focused on the neural targets of these emotional and cognitive responses may be part of the patients’ care in people with long-term diseases.

## Supplemental Material

sj-docx-1-hpq-10.1177_13591053241233509 – Supplemental material for I don’t feel sick: Cognitive and affective processing of self-health associations using the Implicit Association Test Supplemental material, sj-docx-1-hpq-10.1177_13591053241233509 for I don’t feel sick: Cognitive and affective processing of self-health associations using the Implicit Association Test by Eda Tipura, Isabele Jacot De Alcantara, Amélie Mantelli, Léa Duong Phan Thanh, Anna Fischer, Patrik Vuilleumier and Roberta Ronchi in Journal of Health Psychology
