# Supplementary material for: I don’t feel sick: Cognitive and affective processing of self-health associations using the Implicit Association Test
Source: J Health Psychol. 2024 Feb 27;30(1):3–16. doi: 10.1177/13591053241233509 (PMC11686928; doi:10.1177/13591053241233509)
Supplement: sj-docx-1-hpq-10.1177_13591053241233509 – Supplemental material for I don’t feel sick: Cognitive and affective processing of self-health associations using the Implicit Association Test [file sj-docx-1-hpq-10.1177_13591053241233509.docx]

**APPENDIX**

**A - Illustration of the congruent (left) and incongruent (right) conditions in the health-IAT.**


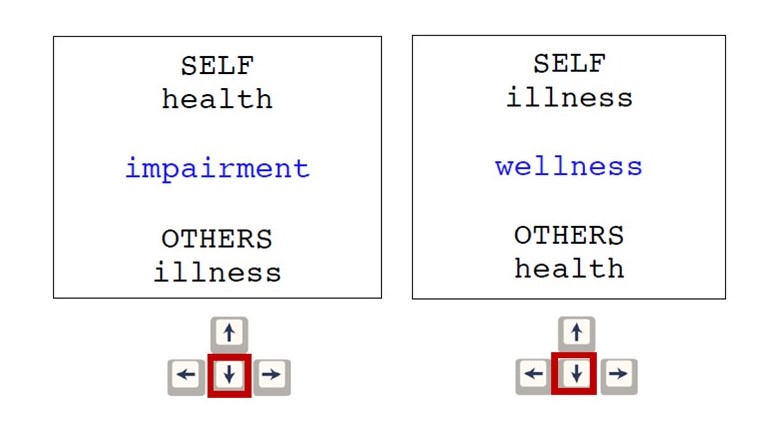


**B - Sequence of trial blocks and associated computer keys with the experimental and control (italics) IAT.**

| Block | | Number of trials | Categories assigned to the computer up-key | Categories assigned to the computer down-key |
| --- | --- | --- | --- | --- |
| 1 | Practice | 20 | Self / *Flower* | Other / *Insect* |
| 2 | Practice | 20 | Health / *Positive* | Illness / *Negative* |
| 3 | Practice | 20 | Self-Health / *Flower-Positive* | Others-Illness / *Insect-Negative* |
| 4 | Test | 40 | Self-Health / *Flower-Positive* | Others-Illness / *Insect-Negative* |
| 5 | Practice | 20 | Illness / *Negative* | Health / *Positive* |
| 6 | Practice | 20 | Self-Illness / *Flower-Negative* | Other-Health / *Insect-Positive* |
| 7 | Test | 40 | Self-Illness / *Flower-Negative* | Other-Health / *Insect-Positive* |

**C - words used in the health-IAT task**

**Self concept words**

MOI

JE

MIEN

MOI-MÊME

NOUS

**Other concept words**

IL

LEUR

ELLES

SIEN

VOTRE

**Health category words**

sain

bien-être

longévité

vitalité

hygiène

énergie

autonomie

équilibre

reposé

bien

bonheur

balade

activité

sourire

profiter

liberté

bénéfique

vigueur

relax

optimal

**Illness category words**

ambulance

amoindri

consultation

déficit

difficulté

diminué

douleur

handicap

hôpital

médicament

perte

prescription

ralenti

réadaptation

risque

séquelle

soins

souffrance

stress

trouble
